# Why Don’t More Smokers Switch to Using E-Cigarettes: The Views of Confirmed Smokers

**DOI:** 10.3390/ijerph14060647

**Published:** 2017-06-16

**Authors:** Neil McKeganey, Tiffany Dickson

**Affiliations:** Centre for Substance Use Research, Block 3/2 West of Scotland Science Park, Glasgow G20 O6P, UK; tiff@csures.org

**Keywords:** e-cigarettes, smoker’s perceptions, reduced harm, smoker’s experience

## Abstract

Whilst e-cigarettes have been characterised by Public Health England as being around 95% less harmful than combustible tobacco products, only a minority of current smokers (around 16% within the UK) are using these devices. In this paper we report the results of an online survey of 650 smokers in contact with a smokers’ rights group in the UK. A total of 91% of the smokers surveyed were smoking on a daily basis. Fifty nine percent reported having used electronic nicotine delivery systems, the majority of whom reported having used e-cigarettes. Those smokers that had not used these devices principally explained this in terms of the pleasure they derived from smoking. The features smokers’ liked most about e-cigarette had to do with the range of settings in which they could be used, the lack of an offensive smell associated with their use, the available flavours and the reduced level of harm. The elements which smokers liked least about e-cigarettes had to do with the vaping experience, the technology, the chemical nature of e-liquids and the complex technology that was associated with these devices. If a greater number of smokers are to be encouraged to take up e-cigarettes, it will be necessary not only to convey accurate information on the relative harm of these devices (compared to combustible tobacco products), but to ensure that they are able to be used in a wider range of settings than those within which smoking can currently occur and that the vaping experience more closely resembles the smoking experience.

## 1. Introduction

In one of the most quoted dicta of international tobacco control, Professor Michael Russell observed in 1976 that “People smoke for nicotine but they die from the tar” [1]. The accuracy of the second half of that statement has been demonstrated by decades of research documenting the serious health harms caused by smoking combusted tobacco [2,3,4]. However, if it is the case that people smoke solely or principally for the nicotine one might have anticipated that the development of alternative, less harmful means of delivering nicotine, would result in a large proportion of smokers rapidly switching to the lower harm producing products. The experience of e-cigarettes, however, which enable the user to inhale nicotine in aerosolized form, and which have been characterised by Public Health England as around 95% less harmful than combustible tobacco products, shows that transitioning smokers away from combustible cigarettes is a good deal more challenging than simply providing an alternative means of consuming nicotine [5].

According to the UK charity “Action on Smoking and Health” (ASH) there are approximately 2.8 million people in Great Britain using e-cigarettes, 51% of whom are current smokers [6]). ASH has also estimated that there are approximately 8.7 million adult smokers within the Great Britain [7]). On the basis of those estimates it would appear that only around 16.4% of adult smokers within the Great Britain are using e-cigarettes. Although these estimates are likely to contain a margin of error, the impression conveyed is that only a minority of smokers have switched to sole use of the reduced risk product.

There is likely to be substantial benefit to the individual, and to society, in encouraging those smokers who cannot quit smoking, to switch to using a non-combustion based means of consuming nicotine. As a result, there is a need to identify the possible barriers to the wider adoption of e-cigarettes by current smokers. In the research we have undertaken we have looked at the views of those smokers who have been somewhat overlooked in research on e-cigarettes to date, namely smokers with a high level of commitment to smoking and who may well see smoking as a core part of their identity. Smokers in contact with a smokers’ rights group are likely to have the strongest attachment to their continued smoking and on that basis could be seen to pose the greatest challenge to those seeking to encourage the wider adoption of e-cigarettes by current smokers. Better understanding the views of these smokers with regard to how they view e-cigarettes, their willingness to use these devices, their experience of these devices, and the possible perceived barriers to their use, may provide insights that are relevant to increasing the wider appeal of e-cigarettes to current smokers.

## 2. Methods

In September 2016, we undertook an online survey of smokers in contact with the UK’s leading smokers’ rights organization (Forest). The invitation to take part in this research was included in an advert placed in the Forest newsletter and circulated to those in contact with the organisation. It is impossible to provide a precise figure on the number of individuals who will have seen the invitation to contribute to this survey although Forest estimates that number to be between 2000 and 2500.

The instrument used in this research took approximately 15 min to complete and included both closed and open ended questions covering the individual’s smoking history, reasons for smoking, the individual’s perceived likelihood of smoking in the future, their willingness to stop smoking, their views of National Health Service (NHS) stop smoking services, and their use and views of new nicotine/tobacco products such as e-cigarettes. In this paper, we principally focus upon the smoker’s perceptions and use of the new nicotine/tobacco products (e-cigarettes). Analysis of the qualitative responses on the use of these products involved reviewing all of the smokers open ended comments reporting on whether they had used these devices, their views of e-cigarettes, their reasons for not using e-cigarettes, what they liked most and least about e-cigarettes, their choices in relation to either continuing or discontinuing to use e-cigarettes, and their views as to how comparable the vaping experience was to smoking. In analyzing qualitative data it is often the case that researchers present data in an illustrative way without conveying the range and frequency of responses around particular topics. In this paper we outline both the content of the smokers’ views and experiences of the new nicotine devices (principally e-cigarettes) and the frequency with which those views were expressed across the sample of smokers surveyed. In addition to presenting qualitative data we also provide some quantitative information on the sample surveyed, the frequency and stated reasons for their smoking, their intentions with regard to continuing or ceasing smoking in the future, and the likelihood of their using e-cigarettes.

## 3. Results

We undertook an online survey of 650 smokers in contact with the Forest organisation. Respondents were aged between 18 and 88 years old (*N* = 581; *M* = 55.64, sd = 13.29), were mainly male (66%), and had smoked for between 1 and 73 years (*N* = 573; *M* = 38.58, sd = 14.21). Most participants smoked daily (91%). Those who did not (*N* = 53) tended to smoke on six (23%), four (23%), or two (13%) days per week [five days per week = 8%; three days per week = 9%; one day per week = 8%; one day every two weeks = 2%; one day every three weeks = 2%; one day per month = 2%], but some smoked less than one day per month (11%). More than three quarters of this population reported that they saw themselves as smoking well into the future (77%) with only 8% indicating that they envisaged a time in the near future or immediately when they would have stopped smoking. Nearly all of the smokers surveyed (95%; *N* = 583) identified enjoyment as their reason for smoking, with 35% indicating that smoking comprised an important part of their self-identity.

Results of linear regression analyses suggest that being younger [β = −0.14, *p* = 0.001] and being male [β = −0.09, *p* = 0.03] predicted higher ratings of enjoyment of smoking. After controlling for the effects of age and gender, the length of time spent as a smoker was significantly predictive of self-reported enjoyment of smoking [β = −0.23, *p* = 0.03] showing those who had been smoking for a longer period of time enjoyed smoking significantly less than newer smokers, regardless of age or gender. To assess smokers reported levels of enjoyment of smoking we used a ten-point scale ranging from “not enjoyable at all” through to “extremely enjoyable”. Independent samples t-tests show that those who had tried any of the new nicotine products such as e-cigarettes reported enjoying cigarettes significantly more (*M* = 2.21, sd = 1.58) than those who had not (*M* = 1.90, sd = 1.50) [t(541) = 2.13, *p* = 0.03]. Those smokers who felt that they might switch to any of these products in the future reported significantly greater enjoyment of cigarettes (*M* = 3.12, sd = 1.99) in comparison with those who did not expect that they would ever switch (*M* = 1.74, sd = 1.34) [t(79.43) = 4.97, *p* < 0.001].

When it came to trying any of the new nicotine delivery products there was no statistically significant difference between participants with experience of smoking-related ill health. However, of the participants who had tried one of these new products, those with a health problem attributed to smoking were significantly more likely (yes = 21%, no = 32%, maybe = 47%) than those without a health problem caused by smoking (yes = 14%, no = 48%, maybe = 37%) to answer maybe or yes to the question, ‘Do you think you might switch to any of these products in the future?’ [χ^2^(2)= 8.20, *p* = 0.02].

## 4. Smokers Views of E-Cigarettes

In total, 344 (59%) of those who responded to our questionnaire provided information on their use of reduced risk nicotine products. Almost all of those (336) reported that they had used e-cigarettes. We look first at the smokers’ reasons for not having even tried any of the available alternative nicotine delivery systems. The most commonly cited reason smokers offered for not having tried any of the available electronic nicotine delivery systems was that the individual enjoyed smoking, and was not interested in using a device which, in their view, was associated with quitting smoking/tobacco. In total, 91 of the 114 smokers who commented that they had not used any of the electronic nicotine delivery systems explained their non-use by referring to their preference for smoking and their decision to continue smoking. Comments such as the following were typical in this regard:

Because I enjoy smoking. There seems to be an assumption that every smoker wants to give up smoking. Whilst this is true for some it is not true for all smokers.

*(59 years old female smoker)*

E-cigarettes do not appeal to me because they have no tobacco in them. I do not think that I derive enjoyment from the nicotine alone. I think there are other substances in tobacco that are beneficial and enjoyable besides the nicotine.

*(47 years old male smoker)*

Other, less commonly cited, reasons for not having even tried e-cigarettes were a perception that the devices were unreliable, that the batteries used within e-cigarettes often caused problems, that the devices were too complicated, and that they might be associated with long-term health harms that may only become evident in the future. In total, 23 of the smokers who said that they had not used e-cigarettes drew attention to these further reasons for non-use. Comments such as the following were typical:

They don’t appeal to me. I suspect they wouldn’t taste the same and that they’d be a poor substitute. Besides I’ve read about them and they seem very complicated.

*(54 years old male smoker)*

Worse than normal cigarettes. Normal smoking has been around for so long. You know where you are with normal traditional smoking. E-cigarettes have not been tried and tested enough to be flooding the market as they are. I predict there will be campaigns about e-cigarettes in the future. Breathing fluid into your lungs.

*(72 years old female smoker)*

They are awful and nothing like having a real cigarette or roll up and in a few years they will find some new detrimental health condition that e-cigarettes have caused probably worse than the effects of smoking tobacco.

*(58 years old female smoker)*

E-cigarettes are extremely toxic, not natural and far more dangerous than organic tobacco.

*(55 years old female smoker)*

What is very clear in these comments is the fact that those smokers who had chosen not to use e-cigarettes were not looking for an alternative means of consuming nicotine and tended to view e-cigarettes in a largely negative light; seeing them as being less appealing, less satisfying, and in some cases more harmful than combustible tobacco products. In the next section, we look at the views and experiences of those smokers who reported having used e-cigarettes as to what they liked most and least about those devices.

## 5. What Did Smokers Like Most about E-Cigarettes?

In total, 201 smokers commented on what they liked most about e-cigarettes with the largest category of responses (59) having to do with the fact that these devices could be used in a much wider range of settings where combustible tobacco products were typically banned:

I can still do it in most of the places I socialise in.

*(60 years old male smoker)*

I can vape inside my place of work.

*(64 years old male smoker)*

Not having to go outside in the rain to vape.

*(43 years old female smoker)*

The next most frequent set of comments setting out what the smokers liked most about e-cigarettes had to do with the lack of an offensive smell, coupled with the wide range of flavours that could be vaped in e-cigarettes. These comments were mentioned by 51 of the smokers:

No tobacco smell.

*(63 years old female smoker)*

My clothes and hair not smelling like an ashtray and not having to find a lighter.

*(43 years old female smoker)*

No lingering taste or after smell.

*(69 years old female smoker)*

The taste of my vanilla custard is fantastic.

*(33 years old male smoker)*

The next most commonly expressed set of views (34) identifying what the smokers liked most about e-cigarettes had to do with the relative price of e-cigarettes compared to combustibles:

It costs that much less.

*(70 years old male smoker)*

Interestingly, only 19 smokers drew explicit attention to the lower level of harm associated with e-cigarettes as being something that appealed to them:

Healthier all round.

*(64 years old female smoker)*

The fact that it is steam rather than smoke that is given off, therefore could not be accused of endangering the health of others through passive smoking.

*(54 years old male smoker)*

Being able to breathe again. Not having the fear of dying all the time and the fact that it made quitting smoking incredibly easy.

*(56 years old male smoker)*

Finally, a handful of smokers drew attention to the technology of the devices they had used as forming a large part of their appeal:

Fascination with the technology.

*(55 years old male smoker)*

I enjoy the customisation options available in both hardware and flavours.

*(36 years old male smoker)*

It is evident in these smokers’ comments that a large part of the appeal of e-cigarettes had to do with what was seen to be the increasingly restrictive regulation of combustible tobacco products, with e-cigarettes offering a way in which smokers could continue to consume nicotine in settings where they would otherwise not be allowed to smoke. For these smokers, e-cigarettes did not have to be as appealing, or as effective as combustible tobacco products, in delivering nicotine but had to be able to be used in a much wider range of settings (than combustible tobacco products) whilst delivering an acceptable/enjoyable user experience.

Although much has been made of the claim that e-cigarettes are at least 95% less harmful than combustible tobacco products, this issue of the relative harm of combustible and non-combustible products was a less prominent part of the smokers assessment of the appeal of e-cigarettes than the fact that they could be used in a wide variety of settings, with a wide range of flavours, with the lack of an offensive smell, and with vaping being seen as less costly than smoking. It is also interesting that amongst those smokers who commented positively about e-cigarettes, relatively little of that appeal had to do with e-cigarette technology itself which has undergone major changes in recent years with popular vaping devices looking less like normal cigarettes (cig-a-like systems) and more distinctive in their own right (open, customisable systems, with large nicotine tanks and batteries).

## 6. What Did Smokers Like Least about E-Cigarettes?

In relation to what the smokers liked least about e-cigarettes the most commonly expressed criticisms had to do with a perceived shortfall in the technology of the devices themselves (*N* = 53), followed by negative comments about the taste and flavours (*N* = 24) and the sensation of vaping (*N* = 21). A total of 16 individuals drew attention to health concerns about e-cigarettes with the majority of those having to do with individuals coughing following vaping. A small number of individuals commented negatively about what they perceived to be the rather “cliquey” feel of e-cigarettes and e-cigarette culture and the price of equipment. Comments such as the following were typical of those provided by the smokers:

I hate the weight of the appliance and the metallic feel.

*(69 years old female smoker)*

The size and shape of them.

*(67 years old female smoker)*

All the paraphernalia that goes along with vaping batteries, refills etc.

*(69 years old male smoker)*

The feel of hard plastic in my mouth.

*(69 years old female smoker)*

They feel cold compared to my cigarettes.

*(49 years old female smoker)*

Having to carry all the kit around and having to charge the battery.

*(48 years old male smoker)*

It felt artificial and I couldn’t understand all the liquids strengths and paraphernalia like batteries and coils and things.

*(54 years old male smoker)*

The mess of re-filling and the fact that the e-cigarettes don’t last long before having to replace parts and filters.

*(54 years old male smoker)*

Whilst e-cigarettes technology has improved in recent years, with a proliferation of flavours and a wide variety of different devices, on the basis of these smokers’ views there remain major barriers to the wider use of these devices. Repeated attention was drawn to negative assessment of the material with which these devices are manufactured (hard plastic compared to the softer feel of normal cigarettes), the weight of the devices, their size, their “unnatural” taste, the chemical composition of the liquids, the complexity and somewhat specialised knowledge that is associated with using these devices, and the someway “cliquey” character of vaping culture. In the next section we focus on the views of those smokers who were combining their use of combustible and non-combustible products.

## 7. Smokers Reasons for Combining Vaping and Smoking Rather than Switching Exclusive to Vaping

Overall, 107 smokers provided reasons for why they combined vaping and smoking rather than opting to switch to exclusive use of a lower harm product. The most commonly cited reason smokers offered for not switching had to do with the perceived enjoyment which they said they derived from continuing to smoke. This was mentioned by 36 respondents:

Still enjoy smoking.

*(35 years old male smoker)*

Nothing takes the place of smoking. I prefer smoking especially good cigars.

*(74 years old female smoker)*

I have no desire to, I enjoy smoking.

*(65 years old female smoker)*

The next most commonly offered reason for not having adopted vaping as an exclusive alternative to smoking was that the experience of vaping was not as pleasurable as smoking. This was mentioned by 27 smokers:

Vaping is only a temporary substitute for smoking but it’s not sufficient to replace it outright. The real answer should be more Research and Development on safer combustible cigarettes.

*(56 years old male smoker)*

Not the same pleasure or effect.

*(43 years old female smoker)*

Didn’t enjoy it. Vaping is not quite there yet as a substitute. Real cigarettes are more satisfying.

*(44 years male old smoker)*

Alongside these frequently cited main reasons for combining vaping and smoking, a minority of smokers also cited the fact that they had experienced adverse reactions to vaping and as a result had been reluctant to switch entirely from combustibles to vaping:

Vaping caused me to cough and get a sore throat.

*(70 years old female smoker)*

Made me feel ill.

*(61 years old female smoker)*

Harsh on the throat. Makes you cough.

*(49 years old male smoker)*

A small number of smokers emphasised that in their view, hitherto unknown health concerns about vaping might emerge in the future that would prove these devices to be more harmful than currently thought. For these smokers, whilst the enormous harms associated with smoking were uncontested, the fact that those harms were known about, in contrast to the lack of certainty about the possible long terms harms of vaping, seemed somehow to make smoking more acceptable than vaping:

I am not convinced that there aren’t any long-term health risks with vaping. At least with smoking cigarettes I am aware of the chemicals I am breathing in.

*(54 years old female smoker)*

A small number of smokers explained their reticence to use vaping technology on anything other than an occasional basis in terms of their views about the technology itself, what it looked like, how it functioned, and how people reacted to their being seen vaping:

It looks like the clay pipes the last generation used to smoke. Personally I think it looks ridiculous smoking looks much more glamorous.

*(67 years old female smoker)*

They can be unreliable, battery runs low or fluid runs out.

*(49 years old male smoker)*

A handful of the smokers surveyed, who were dual using e-cigarettes and combustibles, commented that in their view vaping was a poor substitute for what was regarded as the “real thing” of tobacco smoking:

I’ve got my doubts about it not being natural but completely artificial. Having grown tobacco for a couple of years now I know the plant. I trust plants. I don’t trust chemicals.

*(67 years old male smoker)*

I prefer the real thing.

*(67 years old male smoker)*

I mistrust the vaping products and I do not like the experience and I will not be cajoled into using a synthetic alternative.

*(56 years old female smoker)*

I prefer the authenticity of smoking a real cigarette as the taste and experience on the whole is more original and thus enjoyable.

*(43 years old male smoker)*

Whilst there was a clear sense in these comments that smoking remained their preferred activity, nevertheless a minority of smokers commented that they regarded vaping as a way in which they could reduce the frequency of their smoking:

I do both because this allows me to cut down on the quantity of cigarettes I smoke but allows me to continue to enjoy a proper cigarette when I want one.

*(67 years old female smoker)*

I enjoy both. I smoke much less than I used to before vaping and am happy with the current balance.

*(42 years old male smoker)*

Finally, in terms of the perceived negative factors that moderated the smokers’ enjoyment of e-cigarettes, and which may have explained their partial adoption of the devices, there was a sense of embarrassment that some individuals felt at being seen using these devices in public:

I feel a bit embarrassed using them in public.

*(41 year old male smoker)*

With regard to what was principally driving vaping amongst these dual users it was clear that the most influential factor was the greater range of settings within which e-cigarettes could be used compared relative to the many settings in which smoking is prohibited:

I only use them as an alternative when cigarettes are not able to be smoked such as when one is on a train or plan or in airports where there are no facilities.

*(65 years old male smoker)*

They’re a means to allow me to pass through severe smoker hostile places.

*(36 years old female smoker)*

I only vape or use gum when travelling so airports and planes for gums, e cigs I use when forced to use in a non-smoking hotel or in the loo when an outpatient at hospital or in the loo on trains.

*(44 years old male smoker)*

In contrast to the frequent comments that explained their use of e-cigarettes in terms of the wide range of settings within which the devices could be used, only a very small number of smokers explained their use of e-cigarettes in terms of their nicotine dependence.

## 8. Discussion

On the basis of previous research it is evident that despite their lower harm profile (compared to combustible tobacco products) the uptake of e-cigarettes by current smokers has been relatively modest. For example, according to the UK anti-smoking charity “Action on Smoking and Health” only 16.4% of adult smokers within the Great Britain are using e-cigarettes [6]. There are likely to be a range of reasons why e-cigarettes have not proved to be as attractive to current smokers as one might have hoped. One possible reason might be that these devices are less effective in delivering nicotine to the user in comparison to combustible tobacco products. Smoking combusted tobacco results in nicotine reaching the users’ brain within 10 to 20 s after inhalation [8]. Whilst early generation e-cigarettes were less effective in terms of the speed with which the user felt the effects of nicotine inhalation, more recent developments in vapor product technology has meant that these devices are now much more effective in terms of the speed with which they deliver nicotine to the users’ brain [9,10,11].

Alongside the capacity of e-cigarettes to deliver a user-experience that closely approximates smoking, there are a range of other factors that may impact upon the likelihood of smokers’ switching to reduced harm products including variations in the price of these devices in comparison to the price of combustibles [12,13]; critical media and scientific comment on the use of e-cigarettes [14,15]; the operation of local banning orders restricting the use of e-cigarettes in certain locations [16]; the reactions of other people to the visible use of these devices and whether they are seen as stigmatising [17]; and the perceptions of these devices by smokers and others [18].

Whilst many studies have reported smokers’ perceiving e-cigarettes to be less harmful than combustible tobacco products there has been a notable increase in the perception that these devices might be more harmful than combustible tobacco products. For example, Majeed and colleagues [19] have reported data from the U.S. on perceptions of the relative harms of e-cigarettes and normal cigarettes which suggests that this might indeed be the case. In the Majeed et al. study the proportion of current smokers who believed e-cigarettes were less harmful than combustible tobacco products fell from 44.7% in 2012 to 36.0% in 2015, whilst the number who believed that e-cigarettes were more harmful increased from 0.7% to 4.3%. On the basis of their findings Majeed and colleagues concluded that:

Higher risk perceptions of e-cigarettes could deter current smokers from using e-cigarettes as a cessation aid of smoking combustible cigarettes and preventing a potential public health benefit.

*[19] (p. 335)*

The findings from the Majeed et al. study echo similar research from the UK. According to the Office for National Statistics, amongst current and ex-smokers in Great Britain who had never used an e-cigarette, 23.1% perceived e-cigarettes to be as harmful as combustible tobacco products whilst 7.2% thought that e-cigarettes were more harmful or much more harmful than combustible tobacco products of [20]. Similarly, Brose and colleagues reported a decreasing proportion of smokers perceiving e-cigarettes to be less harmful than combustible tobacco products over the period 2013 and 2014 [21]. Despite the increase in the proportion of people perceiving e-cigarettes to be more harmful than combustible tobacco products it is still the case that the predominant view of these products is that they are safer than normal cigarettes.

Glasser and colleagues [18] have identified a total of 188 articles reporting data on consumers’ perceptions of e-cigarettes and other electronic nicotine delivery systems. Consumers’ reason for using electronic nicotine delivery systems included the perception that these devices were less harmful than combustible tobacco products [22,23]; that they offered a means of quitting or reducing smoking [24,25,26]; and that they offered a means for evading smoke free policies and of avoiding the impact of second hand smoke [27,28].

On the basis of the various studies undertaken it is clear that the use and perception of e-cigarettes varies significantly across different social and demographic groupings. For example, Brose and colleagues [21] have shown that older smokers, and those who had stopped smoking were more likely than younger current smokers to perceive e-cigarettes as being less harmful than combustible tobacco products. Fallin et al. [29] looked at e-cigarette use amongst pregnant and post-partum women. In this small, qualitative study (*N* = 12), the researchers found that whilst the women were initially attracted to e-cigarettes as a way of reducing their level of smoking, once their baby was born there was a tendency to revert to smoking combustible cigarettes. The researchers on this study also noted that the advice from health care providers in relation to the women’s e-cigarette use was highly variable with some health care providers actively discouraging the use of e-cigarettes. Bagget et al. [30] looked at e-cigarette use amongst a sample of 306 homeless adult smokers’. Amongst respondents in this study, 24% had used e-cigarettes with curiosity being the main reason for such use. The authors on this study encourage health care workers in contact with homeless people to discuss possible e-cigarette use with their clients. Pratt et al. [31] looked at e-cigarette perceptions and use amongst smokers with serious mental illness. In this small study (*N* = 19) smokers were provided with e-cigarettes over a four-week period. Whilst respondents were positive in their assessment of these devices they tended to use them alongside rather than in place of combustible tobacco products. Even so the provision of e-cigarettes to study participants was associated with a notable reduction in the rate of smoking with the number of cigarettes per week consumed falling from an average of 192 to 67 over the four-week study period.

In the research we have undertaken we looked at the views and experiences of one group of smokers, namely those who are committed to smoking: 91% of our sample were daily smokers, 77% saw themselves as smoking well into the future, 35% regarded smoking as being part of their core identity, and 95% cited enjoyment as their principal reason for smoking. The group of smokers we have surveyed provided information via an online survey advertised to those in contact with a smokers’ rights group. There is no sense in which this group should be regarded as being representative of smokers in general. These are individuals who, in their commitment to smoking, are about as far from the public health perspective of seeking to reduce or end smoking as it is possible to get. They are also a group who, in their continued smoking, have shown themselves to be somewhat immune to any of the current initiatives aimed at encouraging smokers to quit. In seeking to understand why the uptake of e-cigarettes has been so modest amongst current smokers it is helpful to consider the views and experiences of these smokers for the very reason that they represent, in their very extremity, the challenge which those in tobacco control and public health face in trying to encourage more smokers to take up e-cigarettes as a less harmful alternative to combustible tobacco products [32].

Given that so few of the smokers we surveyed indicated a commitment to quitting smoking, and the fact that e-cigarettes are typically presented as an aid to quitting smoking, one might have anticipated that relatively few of our smokers would have been interested in these devices. In fact, however, over half of our respondents reported having used e-cigarettes. This shows that even amongst this group of confirmed smokers there is a willingness to at least try alternatives to combustible tobacco products. This is important because previous research has shown that even amongst those smokers with no interest in quitting there can be benefits in individuals using e-cigarettes. Polosa et al. [33], for example, looked at the use of e-cigarettes amongst smokers not intending to quit. In this study, 50 smokers who had no prior commitment to stopping smoking were provided with e-cigarettes over a 6-month period. Whilst they were encouraged to use these devices they received no additional encouragement regarding smoking cessation and were told that they were at liberty to smoke their own cigarettes alongside the e-cigarettes provided to them if they so wished. Remarkably, the researchers report an 80% reduction in average cigarettes per day consumption (25 to 5) over the study period leading them to conclude that even for smokers not intending to quite there are benefits in being encouraged to use e-cigarettes.

Amongst the smokers we surveyed, e-cigarettes were principally seen as offering an additional means of consuming nicotine rather than as a sole replacement to combustible tobacco products or a way of quitting smoking. The smokers who reported positive views of e-cigarettes highlighted the greater range of settings in which e-cigarettes could be used, the variety of the flavours that were available, the lack of an offensive smell associated with their use, their reduced price compared to combustible tobacco products, and the fact that they were associated with less harm to those using the devices and those in the vicinity of such use. Amongst the smokers who highlighted negative views about e-cigarettes attention was drawn to the fact that the vaping experience was seen as less enjoyable than the smoking experience, the possibility that vaping might in time be associated with some hitherto unknown long term harm, at the hard plastic material used in the construction of e-cigarettes, at their weight which meant they could not be comfortably held between the lips in the same was as a cigarette, at the complex and at times confusing technology involved in e-cigarettes, and at the somewhat “cliquey” feel of the culture around vaping.

## 9. Conclusions

If public health agencies are going to succeed in increasing the percentage of smokers who are using e-cigarettes, it will be necessary to overcome the various barriers to the wider use of these devices. Whilst much of the current public health information around e-cigarettes has focussed on their lower level of harm (compared to combustible tobacco products) it was notable that the relative harm of these products was not a prominent feature in our smokers’ reasons for why they had used these devices, nor in their reasons for continuing to smoke. If e-cigarettes are going to appeal to a much wider range of smokers, it will be necessary for the vaping experience to be at least as enjoyable as smoking (in terms of smokers’ perceptions) and very probably more enjoyable than smoking. There is an important need to ensure the continued availability of a wide range of flavours and of a wide range of e-cigarette “kit” encompassing technology which is relatively simple and easy to use (cig-a-like) and that which is more complex and appealing to those who enjoy new technology. There is a need to ensure that that these devices can be used in a wide range of public settings without users experiencing the stigma that is sometimes attached to their use [34]. It will require the continued availability of e-cigarettes at a price that makes them competitive with combustible tobacco products, and it will require the vaping experience to be as similar as possible to the smoking experience (in the speed of nicotine delivery, in the effect on the throat, taste, sensation).

In addressing these issues, there is a role here for both manufacturers and health promotion agencies. It is clear that the uptake of e-cigarette use can be influenced by local, national and international legislation. Whilst it was evident in our own research that the imposition of smoking bans was influencing smokers’ willingness to use e-cigarettes, it is equally likely that the extension of those bans to include e-cigarettes which is occurring in some areas will see the use of these devices, and their potential public health benefit, reducing. It is a concern that studies have shown an increasing proportion of smokers perceiving e-cigarettes to be as harmful as combustible tobacco products, and in some cases as being even more harmful than combustible tobacco products. This would suggest that there is a need for public health agencies to do more by way of informing smokers as to the relative risk of vapour and combustible tobacco products. However, if through media [14], professional [35] and scientific reports [36] e-cigarettes are increasingly seen as a threat to public health, it is likely that there will be less willingness on the part of health agencies to encourage the use of these devices amongst smokers who are reluctant to quit, with the result that the potential public health benefit of e-cigarettes will be reduced not enhanced.
